# dbPepNeo: a manually curated database for human tumor neoantigen peptides

**DOI:** 10.1093/database/baaa004

**Published:** 2020-02-22

**Authors:** Xiaoxiu Tan, Daixi Li, Pengjie Huang, Xingxing Jian, Huihui Wan, Guangzhi Wang, Yuyu Li, Jian Ouyang, Yong Lin, Lu Xie

**Affiliations:** 1 School of Medical Instrument and Food Engineering, University of Shanghai for Science and Technology, No. 516, Jungong Road, Yangpu District, Shanghai 200093, China; 2 Shanghai Center for Bioinformation Technology, Shanghai Academy of Science and Technology, No. 1278, Keyuan Road, Pudong New District, Shanghai 201203, China; 3 Key Laboratory of Carcinogenesis and Cancer Invasion, Ministry of Education; Key Laboratory of Carcinogenesis, National Health and Family Planning Commission, Xiangya Hospital, Central South University, Central South University, No. 932, South Lushan Road, Yuelu District, Changsha 410083, China; 4 College of Food Science and Technology, Shanghai Ocean University, No. 999, Hucheng Ring Road, Pudong New District, Shanghai 201306, China

## Abstract

Neoantigens can function as actual antigens to facilitate tumor rejection, which play a crucial role in cancer immunology and immunotherapy. Emerging evidence revealed that neoantigens can be used to develop personalized, cancer-specific vaccines. To date, large numbers of immunogenomic peptides have been computationally predicted to be potential neoantigens. However, experimental validation remains the gold standard for potential clinical application. Experimentally validated neoantigens are rare and mostly appear scattered among scientific papers and various databases. Here, we constructed dbPepNeo, a specific database for human leukocyte antigen class I (HLA-I) binding neoantigen peptides based on mass spectrometry (MS) validation or immunoassay in human tumors. According to the verification methods of these neoantigens, the collection of peptides was classified as 295 high confidence, 247 medium confidence and 407 794 low confidence neoantigens, respectively. This can serve as a valuable resource to aid further screening for effective neoantigens, optimize a neoantigen prediction pipeline and study T-cell receptor (TCR) recognition. Three applications of dbPepNeo are shown. In summary, this work resulted in a platform to promote the screening and confirmation of potential neoantigens in cancer immunotherapy.

Database URL: www.biostatistics.online/dbPepNeo/.

## Introduction

Neoantigens represent a class of short peptides that are derived from tumor-specific somatic mutations. Notably, they can bind to HLA molecules and present on the cell surface, subsequently to be recognized by T-cell receptors (TCRs) to activate the immune system to attack specific cancer cells (1–3). In addition, as neoantigens are rarely expressed in normal tissue cells to bypass central thymic tolerance, they are likely to generate robust immune responses (4). Therefore, they are considered as important targets for development of personalized vaccines (5,6). The promising neoantigen vaccines should be presented to the surface of tumor cells, and they should be recognized by T cells (7,8). Both characteristics are associated with major histocompatibility complex (MHC) binding; therefore, the ability to bind MHC is a necessary precondition of antigenic peptide. The human forms of the complex are known as HLA class I (HLA-I) and HLA class II (HLA-II). Therein, HLA-I molecules can bind to endogenous antigens with the length of 8–11 amino acids and present antigens to the cytotoxic CD8^+^ T cells (9). HLA-II molecules can bind to exogenous antigens with the length of 11–20 amino acids and present antigens to the helper CD4^+^ T cells (10). Here, we only focus on tumor neoantigens, a type of endogenous antigen, which are peptides produced by genomic mutations that are translated, processed and presented by the tumor HLA molecules (11). In addition, TCR recognition to HLA peptides is necessary as only about 1% of the predicted candidate neoepitopes can be recognized by T cells in tumor patients (12). Peptides are usually validated by the reactivity of T cells in peripheral lymphocytes or autologous tumor-infiltrating lymphocytes using ELISPOT or flow cytometry method of tetramer staining (13,14). Neoepitopes are able to be recognized by CD8^+^ T cells, leading to tumor regression after immunotherapy.

In 2017, two independent reports published in *Nature* showed that neoantigen vaccines achieved active efficacy in the treatment of malignant melanoma. Moreover, their efficacy could be further improved by combination with checkpoint immunotherapy of programmed cell death-1 (PD-1) (15,16). The studies demonstrated the potential to develop personalized treatments for cancer. Subsequently, Keskin *et al*. successfully applied personalized neoantigens targeting vaccines to immunize patients newly diagnosed with glioblastoma (17). These studies, both alone and in combination with checkpoint therapies, provide a strong rationale for further development of immunogenic personal neoantigen vaccines. Given the high complexity of HLA polymorphisms and the diversity of HLA ligands, mass spectrometry (MS) has been established as being useful in helping develop neoantigen vaccines (18). Whole-exome sequencing (WES) was reported to combine with HLA peptidomes to identify neoantigens in melanoma patients (19,20), demonstrating that proteomics MS analysis and screening can improve the accuracy of neoantigen prediction based on genomics data and narrow the scope of subsequent immune verification.

To date, several immune peptide databases have been developed, such as Immune Epitope Database (IEDB) (21), TSNAdb (22) and Cancer Immunity Peptide Database (23). IEDB was widely considered as the gateway to global immune epitope information, storing significant specific immune epitope information. TSNAdb contains the neoantigens predicted by NetMHCpan (24) based on somatic mutations of The Cancer Genome Atlas (TCGA) tumor samples and their corresponding HLA allele data of The Cancer Immunome Atlas (TCIA), as well as the experimentally verified neoantigens in IEDB. In the Cancer Immunity Peptide Database (23), a total of 403 tumor antigenic peptides are included, which are classified into unique antigens, tumor-specific antigens, differentiation antigens and overexpressed antigens according to their expression patterns.

For researchers, it is difficult to access the specific data for human tumor neoantigens from experimental validation, because they are scattered among research articles and databases. Therefore, we constructed a manually curated database, named dbPepNeo, in which we collected experimentally supported human tumor neoantigens. The experiments include MS-purposed HLA-I binding peptide detection and the up-to-date immunogenicity verification by specific T-cell response assays. They are categorized according to experimental validation methods: (i) low confidence (LC) neoantigens that were examined by MS; (ii) medium confidence (MC) neoantigens which contain somatic mutations and were verified by MS and WES/whole-genome sequencing (WGS); (iii) high confidence (HC) neoantigens, the immunogenicity of which were directly validated by specific T-cell response experiments. This database provides a basic foundation for further screening of neoantigens, optimization of prediction pipelines and study of TCR recognition.

## Materials and methods

### Data collection and processing

#### Data source and collection criteria

In dbPepNeo, tumor neoantigens bound by HLA-I were extracted from the peer-reviewed neoantigen articles and the existing public data repositories. The procedure of data collection is as follows.

(i) We first searched PubMed database using ‘neoantigen’, ‘tumor’ and ‘cancer’ and curated the resulting publications. Then, we specifically searched publication of neoantigens related to MS in PubMed database using a list of keywords as ‘neoantigen’, ‘neoepitope’, ‘mass spectrometry’, ‘peptidomes’ and ‘peptidomics’. Publication dates was restricted from January 2008 to December 2018, and the species was restricted as humans.

(ii) We searched neoantigen-related databases and collected positive peptides from IEDB and Cancer Immunity Peptide Database. After removing redundant immunogenic HLA-I peptides, we re-integrated all peptides in a standardized format.

After manually extracting peptides from research articles, further filtering was processed according to the neoantigen collection criteria. These inclusion criteria were based on several key neoantigen presentation steps as follows (23,25):

(i) Peptides were isolated from human tumor tissues or cell lines.

(ii) Peptides contained non-synonymous mutations in amino acid sequence.

(iii) Peptides can be bound by HLA-I molecules.

(iv) Peptides can induce CD8^+^ T cell responses.

#### Classification of neoantigens

**Figure 1:**
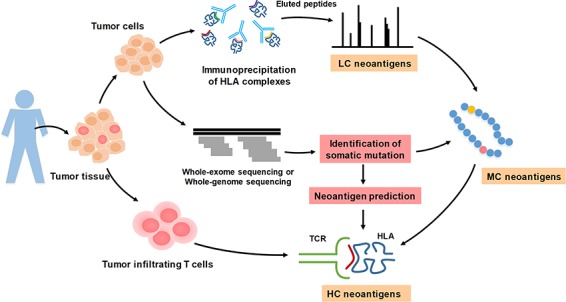
The illustration of high confidence, medium confidence and low confidence neoantigens based on validation approaches.

We summarized several neoantigen-validation methods, and the collected peptides were classified into three categories based on their degree of confidence. The illustration of HC, MC and LC validation methods of neoantigen is shown in Figure 1. First, tumor cells and tumor-infiltrating T cells are extracted from human tumor tissues. Then, HLA complexes are extracted from tumor tissues for immunoprecipitation, and peptides are eluted for further MS analysis. Meanwhile, somatic mutations of tumor cells are identified by WES or WGS. The mutations combined with MS analysis can be used to identify peptides that are presented by HLA-I molecules. On the other hand, the mutated peptides also can be used to predict neoantigens. Next, immunogenicity of mutated peptides can be validated by reactivity to the patient’s tumor-infiltrating lymphocyte. Various experimental verification methods show different stringency and accuracy. T-cell response assay is a high-confidence verification method; in contrast, MS is a low-confidence verification method. Therefore, we defined the raw peptides identified by MS and bound by HLA-I molecules as LC neoantigens; the peptides containing somatic mutations and confirmed by MS and WES/WGS were defined as medium confidence (MC) neoantigens; the immunogenic peptides validated by specific TCRs recognition were considered as high confidence (HC) neoantigens. The LC and MC neoantigens are treated as potential neoantigens and await to be further identified, while HC neoantigens can be selected for developing therapeutic vaccines.

#### Data annotations

According to the data contained in the research articles, we manually annotated neoantigens, including HC neoantigens and MC neoantigens. The information contains cancer type, gene name, HLA allele, mutated peptide sequence, wild type peptide sequence, peptide length, mutation, methods of verification and PubMed ID, as well as the reference links. We also added the mutated peptide affinity IC50 (nM), %Rank and binding level using NetMHCpan (v4.0). According to the Rank% values, the binding affinities were scored and defined: Rank% < 0.5 as strong binding, 0.5 < Rank% < 2 as weak binding and Rank% > 2 as nonbinding. For LC neoantigen datasets, we supply the information as follows: cell line or tissue, number of peptides, URL, identifier, PubMed ID and the reference links.

### Database implementation

**Figure 2:**
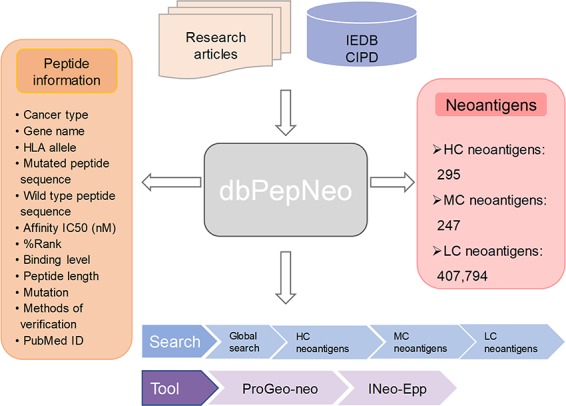
Architecture of dbPepNeo. CIPD: Cancer Immunity Peptide Database; LC: low confidence; MC: medium confidence; HC: high confidence.

dbPepNeo operates entirely using open-source software. The web interface of dbPepNeo was constructed in standard HTML/JavaScript/CSS using the Bootstrap framework as the front end. The back end was written in PHP, connecting the web interface and Apache web server. MySQL was used for data storage. The architecture of dbPepNeo database is shown in Figure 2.

### Sequence similarity analysis

To test the credibility of HC neoantigens and implement the filtering function of dbPepNeo, we used the Basic Local Alignment Search Tool (BLAST) (26) for sequence similarity analysis. HC neoantigens were used to build the target sequence database, while candidate neoantigens were treated as retrieval sequences. Then, BLASTp was used to identify homologous sequences and the degree of homology between candidate neoantigens and HC neoantigens. To increase the sensitivity of BLASTp in short sequence searches, we adjusted the expected value threshold to ‘20 000’. We customized the output as ‘format 6’, parameters of this format include query accession, aligned part of query sequence, subject accession, aligned part of subject sequence, expect value, alignment length and percentage of identical matches. The peptides were reported to have sequence identity to HC neoantigens if the percentage of identical matches was above 60%.

## Results

### Database content

In dbPepNeo, after manual retrieval and mining of the research articles and databases, the immunopeptides verified by low-throughput experiments, as well as by batch production, were extracted. First, 586 neoantigen-related articles and 68 MS-related articles were separately searched by keywords. Because our data collection focused on experimentally verified and MS-screened human neoantigens, with stringent criteria, and after careful filtering, we found that only 33 and 10 articles currently contained the positive data we required, respectively. Consequently, 240 HC neoantigens from neoantigen-related articles were obtained, and 96 HC neoantigens were collected from IEDB and Cancer Immunity Peptide Database. The HC neoantigens from different sources are shown in Figure S1. Overall, we collected 295 HC neoantigens after deleting duplicate peptides (Table S1). The rest of HLA-I binding peptides from high-precision MS data were divided to two parts according to their validation methods, including 247 MC neoantigens (Table S2) and 407 794 LC neoantigens (Table S3).

### Statistics of the collected peptides

**Figure 3:**
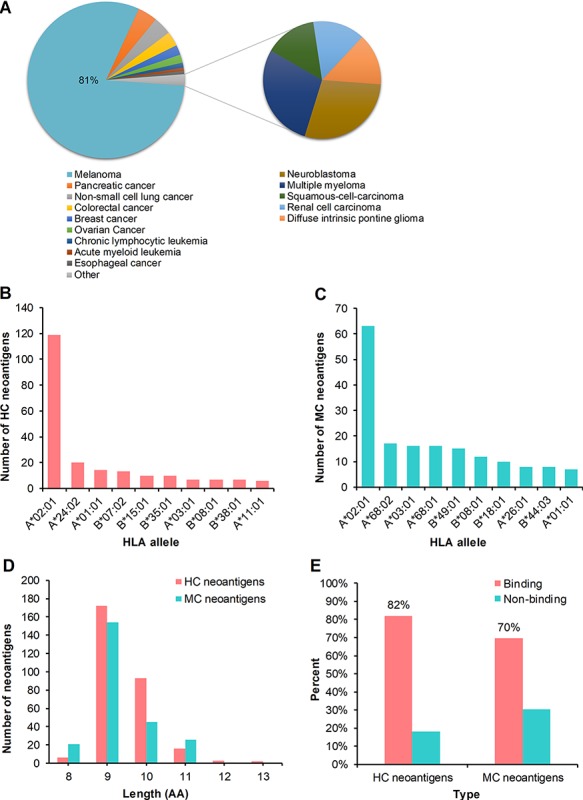
Statistics analysis of the collected peptides in dbPepNeo. (**A**) Distribution of high confidence neoantigens. (**B**) Top 10 HLA alleles matched with high confidence neoantigens. (**C**) Top 10 HLA alleles matched with medium confidence neoantigens. (**D**) The number of medium confidence neoantigens and high confidence neoantigens based on the length of amino acid. (**E**) Affinity prediction with NetMHCpan of medium confidence neoantigens and high confidence neoantigens.

We conducted further statistical analysis on the collected peptides. First, the HC neoantigens involved in 14 cancer types, including melanoma, pancreatic cancer, non-small cell lung cancer, colorectal cancer, breast cancer, ovarian cancer, chronic lymphocytic leukemia, acute myeloid leukemia, esophageal cancer, neuroblastoma, multiple myeloma, squamous cell carcinoma, renal cell carcinoma and diffuse intrinsic pontine glioma. However, 81% HC neoantigens were derived from melanoma, suggesting that melanoma has been widely studied and is a tumor with high mutation burden and thus more suitable for personalized immunotherapy (Figure 3A). Then, the MC neoantigens came from several cancer types or cell lines, such as melanoma, non-small cell lung cancer, and HCT 116 cell line. Non-small cell lung cancer accounts for 68% of MC neoantigens, with the largest amount. Non-small cell lung cancer is also a cancer with high mutation burden. Meanwhile, the 10 most frequent binding HLA alleles matched with HC and MC neoantigens are shown in Figure 3B and C, respectively. Also, HLA-A*02:01 accounted for the largest binding proportion in both HC and MC neoantigens. In addition, we counted the separate numbers of HC and MC neoantigens with the length of 8–13 amino acids (Figure 3D) and found that the majority of HC and MC neoantigens are composed of 9 amino acids, which aligned well with previous reports (3). Furthermore, we used NetMHCpan (v4.0) to predict the affinity between HC or MC neoantigens and the corresponding HLA molecules. The results showed that the HC neoantigens and MC neoantigens that can be bound by HLA molecules with high affinity accounts for 82 and 70%, respectively (Figure 3E).

**Figure 4:**
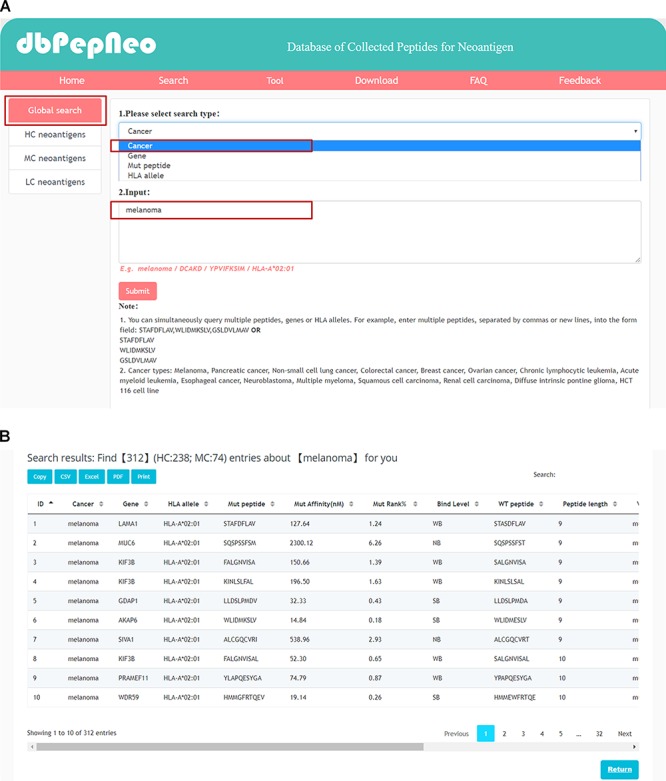
Searching and results presentation in dbPepNeo. (**A**) The workflow of searching in dbPepNeo. (**B**) Results of melanoma under the search of cancer type. Cancer: cancer type; Gene: gene name; HLA allele: HLA allele; Mut peptide: mutated peptide sequence; Mut affinity (nM): mutated peptide affinity IC50 (nM), the predicted binding affinity between mutant peptide and HLA allele by NetMHCpan (v4.0); Mut %Rank: %Rank of mutated peptide, the predicted binding affinity between mutant peptide and HLA allele by NetMHCpan (v4.0); Mut binding level: binding level between mutant peptide and HLA allele; WT peptide: wild type peptide sequence; Peptide length: the number of amino acids contained in the peptide; Mutation: amino acid change; Verification: method of experimental verification; Reference: the supporting literature link; ‘/’: information not provided in the original article is marked as ‘/’.

### Web interface

In order to facilitate retrieval of neoantigens by users in dbPepNeo, we provided a web interface, which comprises of six sections, i.e. **Home**, **Search**, **Tool**, **Download**, **Feedback**, and **FAQ**. On the **Home** page, the introduction and workflow in details are shown. For HC and MC neoantigens, users can click the **Search** box, and then we provide four queries types, i.e. by cancer type, by gene, by peptide and by HLA allele. Also, users can specifically search in **HC neoantigens** or **MC neoantigens** by clicking on **Search** page. In the search results page, we provide a download of data in CSV, excel and PDF formats, as well as ‘copy’ and ‘print’ functions. Also, in this page, users can sort the results by clicking on the table headers. For LC neoantigens, users can click on **LC neoantigens** on **Search** page, and eight data sets links are provided. In the **Tool** page, two neoantigen prediction and study tools developed by our lab are incorporated into dbPepNeo: ProGeo-neo which is a proteogenomics neoantigen prediction pipeline taking use of MS data, INeo-Epp which is a machine learning algorithm for prediction of neoepitope immunogenicity based on the features of neoantigen peptides. In combination with the datasets of HC neoantigens and MC neoantigens in dbPepNeo, the neoantigens predicted by ProGeo-neo can be further classified and screened. In addition, HC neoantigens data can help to optimize INeo-Epp prediction algorithm. All information about neoantigens in dbPepNeo can be freely downloaded by clicking the **Download** button. The detailed user instructions can be found by following the user’s manual in **FAQ** page. Note that users can also send us updated articles about neoantigens using the **Feedback** page to support timely updates of the database.

### Case study 1: four types of queries

dbPepNeo provides four query approaches for retrieving neoantigens: query by cancer type, query by gene symbol, query by peptide sequence and query by HLA allele. As melanoma accounts for the largest proportion in our database (Figure 3A), we use melanoma as an example here. By browsing the melanoma-associated neoantigens (Figure 4A), 312 peptides can be found (Figure 4B). The results show that melanoma is the most widely studied tumor type, possibly due to the high tumor mutation burden of melanoma, which is suitable for immunotherapy. Tumor mutation burden has become a potential biomarker to predict the effectiveness of immunotherapy (27). Additional case searches include an important melanoma-related gene, *DCAKD* (15), which was retrieved by gene symbol, with the result shown in the Figure S2A. A melanoma-related peptide, YPVIFKSIM (15), was retrieved by peptide type, with the result shown in the Figure S2B. Moreover, for the query by peptide type, we presented a fuzzy search method, i.e. peptides can be searched when amino acid sequences are only partially present. In addition, HLA allele is also an important retrieval. We use HLA-A*02:01 as an example, and the retrieval results are shown in Figure S2C. In order to facilitate user retrieval, we provided the function of batch search for querying by gene, peptide and HLA allele. We also supply detailed information on each neoantigen as well as the supporting reference links. Users can select the neoantigens of interest for further analysis and research.

### Case study 2: broad-spectrum filtration of neoantigens

In reality, many factors can influence the prediction of neoantigens in tumors, and the affinity between neoantigens and HLA molecules can only account for some situations. A large amount of false positive peptides may be generated, when only using the prediction algorithm. MS technology was used to improve screening efficiency of tumor neoantigens; however, experimental verification of TCR recognition is essential. The effective peptides can be further screened by our database. In dbPepNeo, the HC neoantigen dataset may be used for high-confidence screening of general solid tumors, thereby reducing the burden of post-experimental validation. We will use the following example to illustrate this application.

A previous study reported 40 shared neoantigens predicted from high-frequency mutations of nine common human malignant solid tumors (including gastric cancer, colorectal cancer, esophageal squamous cell carcinoma, liver cancer, lung adenocarcinoma, lung squamous cell carcinoma, pancreatic cancer, ovarian cancer, and cervical cancer), of which six can generate immune response and induce tumor regression in personalized immune experiments on patients (28). In this case, the 40 shared neoantigens were used to verify the reliability of HC neoantigens in dbPepNeo. We constructed a positive peptide library with 295 HC neoantigens, and then further screened 40 shared neoantigens through the positive peptide library using BLASTp. The results showed that the 24 shared neoantigens were similar in sequence with HC neoantigens, including six neoantigens validated by immunoassay experiments (Table S4). The percentage of identical matches of shared neoantigens and HC neoantigens sequences ranged between 78 and 100%. In general, the possibility of neoantigen recognition by TCRs is proportional to the degree of sequence consistency. Therefore, HC neoantigens can significantly improve the accuracy of neoantigen prediction, providing a broad spectrum of reference for the further screening of general solid tumor neoantigens.

### Case study 3: neoantigen prediction using ProGeo-neo and dbPepNeo

In addition, ProGeo-neo and dbPepNeo together produce a bioinformatics pipeline for mining tumor-specific antigens based on next-generation sequencing, including genomes and transcriptome.

As an example, we used genomic and proteomics data from Jurkat leukemia cell lines to predict neoantigens using the ProGeo-neo pipeline. The results showed that a total of 655 candidate neoantigens were identified. Subsequently, they were further filtered by searching the HC neoantigen library with BLASTp. Nine peptides were found to be consistent with the HC neoantigens sequences (Table S5). The nine mutated peptides may be recognized by TCRs, and their immunogenicity can be further analyzed experimentally or preclinically for leukemia patients. Twenty-two mutated peptides may be found in MC neoantigens; this is still a much smaller range for potential further immunogenicity validation comparing to the original 655 (Table S6).

## Discussion and perspectives

In this study, a comprehensive database, dbPepNeo, is constructed for HLA-I-binding neoantigens based on MS analysis or immunoassay in human tumor. dbPepNeo provides the detailed information about neoantigens of low-throughput experimental verification and high-throughput experiment with enrichment of HLA-I binding high precision MS data, which can help to facilitate further optimization studies and to develop specific targeted neoantigen vaccines. In summary, this work aims at providing a platform to promote the screening and confirmation of potential neoantigens in cancer immunotherapy.

Antigen processing and presentation are complex and involves multiple steps (29). The binding of neoantigens to HLA molecules and the recognition of HLA-peptide complexes to TCRs involve two independent specific binding mechanisms, which result in neoantigen presentation (9). Most of the current neoantigen prediction studies focus on the prediction of the binding affinities between mutant peptides and HLA alleles. Several kinds of integrated software have been developed, such as IEDB, NetMHCpan, PSSMHCpan (30) and SYFPEITHI (31). In dbPepNeo, there exist eight HLA peptide MS datasets (six datasets can be downloaded publicly), which can be used to optimize the prediction pipeline. Also in our database, HC neoantigens causing CD8^+^ T cell responses can be further investigated for the specific recognition of TCRs and HLA-peptide complexes. In contrast, LC neoantigen datasets are experimentally identified peptides that are actually processed and presented by the tumor HLA molecules. Inevitably, it will contain a large number of peptides expressed in non-coding regions, which may be used to develop a neoantigen prediction workflow in the future.

However, there are still many questions to be tackled in this field. For instance, the efficient validation of neoantigens is a primary obstacle to personalized neoantigen-based cancer immunotherapy due to the complexity and technical limitations of immune validation experiments (28). Therefore, the neoantigens verified with high accuracy is limited in size and scope here. Although we have tried to incorporate the most currently identified tumor neoantigens, there is much room for improvement. dbPepNeo will be updated in 2 years, because we anticipate more articles would be published and most experimentally validated neoantigens will still be dispersed in different resources. In the foreseeable future, we would extend our database on the following three key aspects. First, the data verified by MS and immune experiments should be updated. This part will focus on the newly published neoantigen-related articles on PubMed. Second, other functions will be added, such as ‘shared neoantigens’ and ‘driver genes’. In order to widen the application scope of neoantigen vaccine and make it shared among different patients, the study of ‘shared neoantigens’ is bound to become a hot topic, and the hot spot mutation of a strong driver gene in tumors is more likely to express shared neoantigens (28). Third, the non-coding region neoantigens may be included. Laumont *et al*. proved that the non-coding region was the main source of neoantigens, and non-coding regions accounted for 98% of human genome (32), which indirectly explained the poor prediction result of neoantigens in coding regions and the small amount of experimental verification data. However, while the discovery may represent a breakthrough, more experimental research is needed to confirm it. If this conclusion is generalizable, it is believed that when researchers include non-coding regions in the neoantigen prediction, the boundaries and applicability of neoantigens may be further expanded, and universal neoantigen vaccines may become possible.

## Authors’ contributions

L.X. conceived of the idea, planned and coordinated the entire project. L.X., D.L., Y.L. supervised this study. X.J., G.W. and Y.L. contributed to the study design. XT collected and analyzed the data. X.T. and P.H. designed the web interface. P.H. wrote the computer program and constructed the database. H.W. and O.J. helped build the database. X.T. drafted the manuscript; L.X. and X.J. revised the manuscript. All authors read and approved the final manuscript.

## Supplementary Material

Supp_baaa004
