# Supplementary material for: dbPepNeo: a manually curated database for human tumor neoantigen peptides
Source: Database (Oxford). 2020 Feb 22;2020:baaa004. doi: 10.1093/database/baaa004 (PMC7043295; doi:10.1093/database/baaa004)
Supplement: Supp_baaa004 [file supp_baaa004.zip › Supplementary_Figures.doc]

**Supplementary Figures**

**Figure S1.** The overlap of high confidence neoantigens from different sources.

CIPD: Cancer Immunity Peptide Database

**Figure S2.** **(A)** Results of “*DCAKD*” under the search of gene type. **(B)** Results of “YPVIFKSIM” under the search of peptide type. **(C)** Results of “HLA-A*02:01” under the search of HLA allele type.
